# Comparing the Effect of Kangaroo Mother Care on the Serum Bilirubin Level of Term Neonates With Hyperbilirubinemia Under Phototherapy

**DOI:** 10.7759/cureus.81315

**Published:** 2025-03-27

**Authors:** Suraj K Biswas, Utkarsh Bansal, Pragati Sisodia, Ekansh Rathoria, Neeraj K Rao, Nyay B Gupta, Ravindra Ahuja

**Affiliations:** 1 Pediatrics, Hind Institute of Medical Sciences, Barabanki, IND; 2 Pediatrics, Hind Institute of Medical Sciences, Sitapur, IND

**Keywords:** intermittent kangaroo-mother care method, kangaroo care, mother-infant dyad, mother-infant interaction, neonatal indirect hyperbilirubinemia, neonatal jaundice, newborn, side effects of phototherapy, unconjugated bilirubin, unconjugated hyperbilirubinemia

## Abstract

Introduction and objectives

Neonatal jaundice is often treated by phototherapy. Phototherapy is an inexpensive, uncomplicated, and relatively safe treatment option. However, considering certain side effects associated with phototherapy and the resultant mother-infant separation, measures to minimize exposure to phototherapy should be sought. Thus, this study was planned to investigate the effects of combining intermittent kangaroo mother care (KMC) on the duration of phototherapy in neonatal hyperbilirubinemia (NNH).

Materials and methods

It was an observational analytical study. All full-term breastfed infants older than 24 hours of age with bilirubin levels in the phototherapy range were included. The newborns at the time of admission were randomly assigned by simple computer-generated randomization into two groups of 26 cases each (control group and KMC group). In both groups, phototherapy was conducted using standard methods. Infants in the KMC group received intermittent KMC in conjunction with phototherapy. The duration of phototherapy required to treat NNH was the primary outcome.

Results

The infants of the two groups were not significantly different in terms of gender, type of delivery, gestational age, infant's age, mean birth weight, and onset of jaundice, indicating homogenous groups. The median duration of phototherapy was significantly higher in the control group than in the KMC group (p = 0.022). The control group consistently showed higher bilirubin levels than the KMC group: at 24 hours (13.2 mg/dL vs. 11.7 mg/dL, p = 0.047) and 48 hours (11.7 mg/dL vs. 9.9 mg/dL, p = 0.038).

Conclusion

These findings indicate that complementary KMC with phototherapy may reduce the duration of phototherapy in neonatal jaundice.

## Introduction

Unconjugated hyperbilirubinemia occurring in neonates has a huge disease burden worldwide [1,2]. Though this condition is mostly benign, without proper monitoring or treatment, a few cases may develop bilirubin-induced neurologic dysfunction, sensorineural hearing loss, and kernicterus [3].

Phototherapy is commonly regarded as an affordable, simple, and relatively safe treatment for neonatal hyperbilirubinemia (NNH) [4]. However, it has been shown that phototherapy leads to mother-newborn separation along with certain side effects such as water-electrolyte imbalance, temperature instability, bronze baby syndrome, and risk of retinal degeneration and in the long term could be carcinogenic and lead to allergic diseases [5].

Therefore, implementing measures to reduce the duration of phototherapy could be advantageous. Several complementary strategies and treatments have been identified that may help shorten the need for phototherapy exposure, including cycled phototherapy, positioning, use of probiotics, fibrates, metalloporphyrins, zinc sulfate, massage, and kangaroo mother care (KMC) [6]. However, there is no current strong recommendation to use any of the measures. There is a need for studies to investigate and advocate favorable complementary measures that could minimize the duration of exposure to phototherapy without reducing its effectiveness.

KMC has been shown to facilitate a reduction in the incidence of hospital-acquired infections, decrease low-birth-weight infant mortality, and lessen the hospital stay and cost of treatment in preterm infants [7,8], but there is a rising interest in exploring its adaptation to term and late preterm neonates as well. A meta-analysis by Gajula et al. found KMC to be effective in lowering serum bilirubin levels in term and late preterm infants [9]. KMC may help in the management of neonatal jaundice through various mechanisms. On the one hand, it enhances the mother's lactational reflexes, resulting in adequate breastfeeding, which in turn helps lower serum bilirubin levels by improving intestinal motility and accelerating meconium excretion. On the other hand, the vibrations generated by skin-to-skin contact accelerate intestinal excretion, contributing to bilirubin elimination via the digestive pathway. KMC, by alleviating neonatal pain, reduces the amount of energy consumed by newborns due to crying, so more energy is available for increased bowel movements, weight gain, and health recovery, contributing to lower serum bilirubin levels and shortened duration of phototherapy and hospital stay [10].

Intermittent KMC (for short periods once or a few times every day, for a variable number of days) is frequently employed in neonatal intensive care units (NICUs) [11]. We hypothesized that intermittent KMC reduces the duration of phototherapy in term neonates with hyperbilirubinemia. Considering this, the present study aimed to investigate the effects of combining intermittent KMC with phototherapy in reducing the duration of phototherapy in infants with NNH.

## Materials and methods

This observational analytical study was conducted over 18 months at a tertiary care center in North India.

Ethical consideration

Approval from the Institutional Ethics Committee was obtained. A written informed consent was taken from parents.

Inclusion and exclusion criteria

All breastfed infants older than 24 hours of age with bilirubin levels in the phototherapy range according to the American Academy of Pediatrics (AAP) nomogram [12] and with a gestational age of 37-42 weeks were included in the study. Sick neonates requiring NICU admission, those with major congenital anomalies, cephalhematoma, hyperbilirubinemia within 24 hours of life, direct hyperbilirubinemia, prolonged jaundice persisting beyond the third week of life, bilirubin level needing exchange transfusion according to the AAP normogram, Rh incompatibility, hyperbilirubinemia due to G6PD deficiency, history of sibling with neonatal jaundice and with sick mothers unable to breastfeed were excluded from the study.

Sampling and sample size

**Figure 1:**
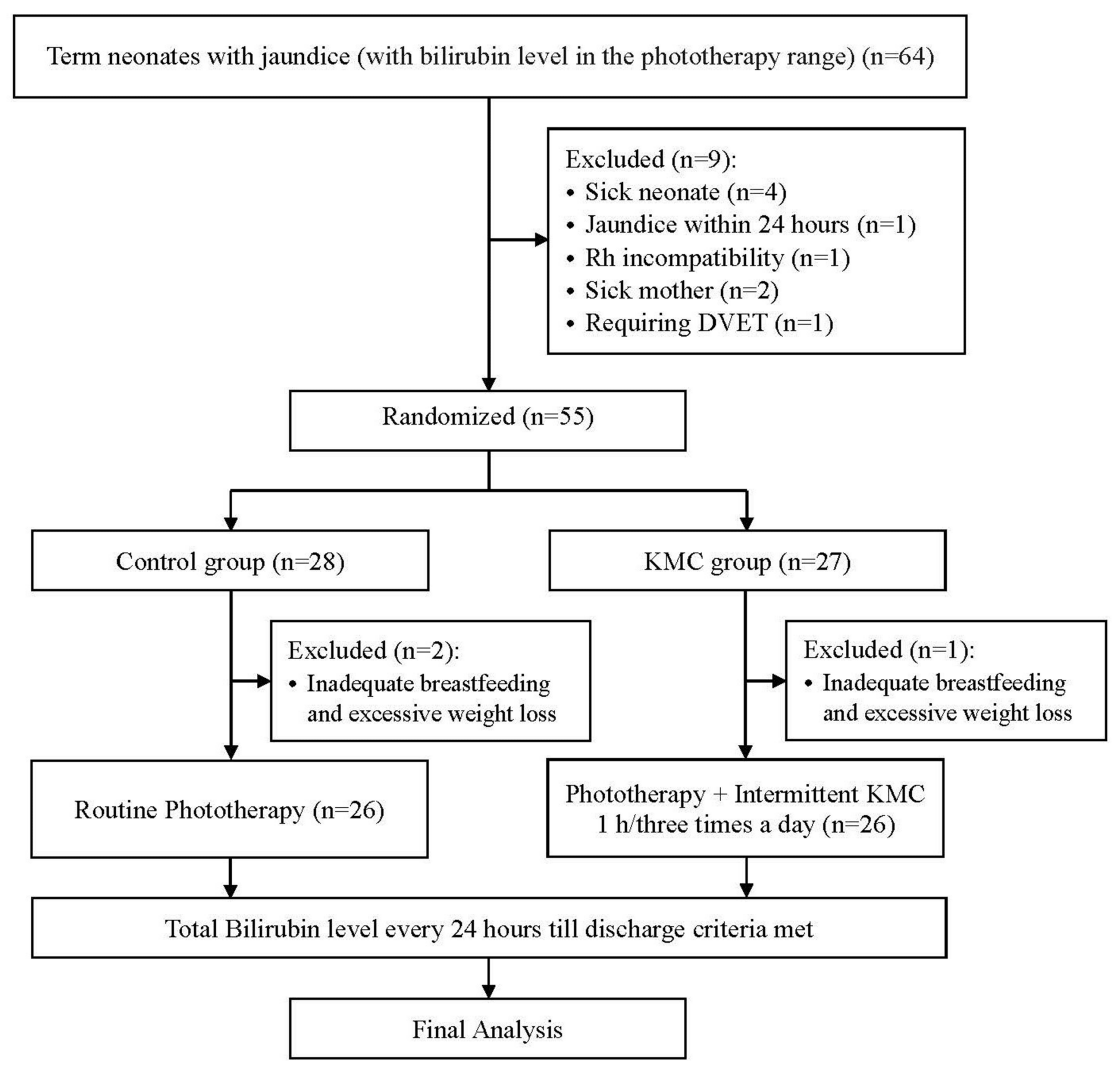
Flow of recruitment of participants in the study KMC: kangaroo mother care; DVET: double-volume exchange transfusion

Considering a previous study [13], employing KMC as a complementary measure to phototherapy, to detect a similar difference (KMC group 68.14 ± 24.32 hours versus control group 100.86 ± 42.26 hours), taking alpha error of 0.05, power of study of 90%, with a confidence interval of 95%, the sample size came out 24 in each arm using the using OpenEpi software [14]. Accounting for 10% attrition, the sample size was increased to 26 in each arm. A total of 55 newborns meeting the inclusion criteria were enrolled in the study. The newborns were then randomly assigned into two groups (control group and KMC group), using a computer-generated randomization table, using simple randomization. Randomization was done using MS Excel (Microsoft Corporation, Redmond, Washington, United States) by applying the appropriate algorithm. Three patients were excluded from the study after enrollment because of inadequate breastfeeding and excessive weight loss. The recruitment procedure is demonstrated in Figure 1.

Methodology

In both groups, neonates received phototherapy using new and equal numbers of lamps providing a light spectrum ranging from 420 to 480 nm, placed at a distance of 35 to 40 cm (Phoenix Brilliance^TM^). Neonates in the control group received routine care and breastfeeding on demand. In the KMC group, neonates received intermittent KMC as 60-minute sessions at least three times daily during the morning, evening, and night shifts after being breastfed, apart from being breastfed on demand. KMC was given in the standard semi-reclined seating position by the mother with the neonate placed between her breasts in an upright position. The mother wore the KMC gown, while the neonate wore a front-open shirt, diaper, and cap. The neonate’s head was kept slightly extended and turned on either side, hips and arms flexed and supported from the bottom with a binder. Mother-neonate dyad was covered with a blanket or shawl during KMC.

The newborns included in the present study were diagnosed and managed according to the AAP guidelines [12]. The weight of the participants was measured daily through an electronic scale (Equinox BE- EQ). The blood specimens were collected at baseline from each infant and were used for the detection of total and direct bilirubin (using diazo method), complete blood cell count, blood group (ABO and Rh), Coomb’s test, G6PD deficiency, and cell morphology by a peripheral blood smear. The mother’s blood group (ABO and Rh) was collected from the postnatal records. The lactation counselor closely monitored and supervised feeding throughout the entire period, ensuring that all neonates received adequate breastfeeding. The newborns were weighed daily, and to further ensure adequate feeding, diaper weight was monitored to check for urine output. Infants who were not breastfeeding adequately despite lactation counseling and showed significant weight loss greater than 3%/day of birth weight were excluded from the study after recruitment. The follow-up total bilirubin was done every 24 hours till it met the discharge criteria. The duration of phototherapy required to treat NNH was used as the primary outcome measure. Other variables, including type of delivery, birth weight, gender, and age after birth, were recorded and compared between the two groups.

Data collection and statistical analysis

The data collected from the cases were compiled in a Microsoft Excel Worksheet and were analyzed using IBM SPSS Statistics for Windows, Version 22 (Released 2013; IBM Corp., Armonk, New York, United States). Descriptive statistics such as frequency, percentage, mean, standard deviation, median, and interquartile range were used to summarize the data. Normality was tested using the Shapiro-Wilk test. Inferential statistics such as the chi-square test, Mann-Whitney U test, and t-test were used to test the significance of differences between groups. A p-value of < 0.05 was considered statistically significant.

## Results

A total of 52 newborns with hyperbilirubinemia requiring phototherapy completed the study, consisting of 25 (48%) males. The etiology of jaundice was “physiological” in 29 (55%) neonates and “ABO incompatibility” in 23 (44%) neonates. Intermittent KMC was given in between sessions of phototherapy to 26 babies (KMC group), while 26 babies received standard care on phototherapy (control group). The infants of the control and KMC groups were not significantly different in terms of gender, type of delivery, gestational age, infant's age and mean birth weight, and onset of jaundice (Table 1).

**Table 1:** Comparison of demographic variables between the two groups KMC: kangaroo mother care; NVD: normal vaginal delivery; LSCS: lower segment cesarean section Data presented as n (%) or ^a^mean (SD), ^b^chi-square value, ^c^t-value. The chi-square and the t-test were used to calculate the p-value. p-value < 0.05 considered significant

Group variable	Control group (n = 26)	KMC group (n = 26)	Statistical test value	p-value
Gender				
Male	14 (53.8)	11 (42.3)	0.642^b^	0.423
Female	12 (46.2)	15 (57.7)		
Birth weight				
<2500 grams	17 (65.4)	15 (57.7)	0.325^b^	0.569
≥2500 grams	9 (34.6)	11 (42.3)		
Mode of delivery				
NVD	16 (61.5)	14 (53.8)	0.3152^b^	0.779
LSCS	10 (38.5)	12 (46.2)		
Cause of jaundice				
Physiological jaundice	14 (53.8)	15 (57.7)	0.078^b^	0.780
ABO incompatibility	12 (46.2)	11 (42.3)		
Mean gestational age^a^ (weeks)	38.69 (0.99)	38.66 (0.98)	0.1127^c^	0.455

The time duration of KMC was three hours in 16/26 (61.5%) neonates, followed by six hours in 6/26 (23.1%) and nine hours in 4/26 (15.1%) neonates in the KMC group, with a mean time of KMC was 4.8 ± 2.0 hours.

**Figure 2:**
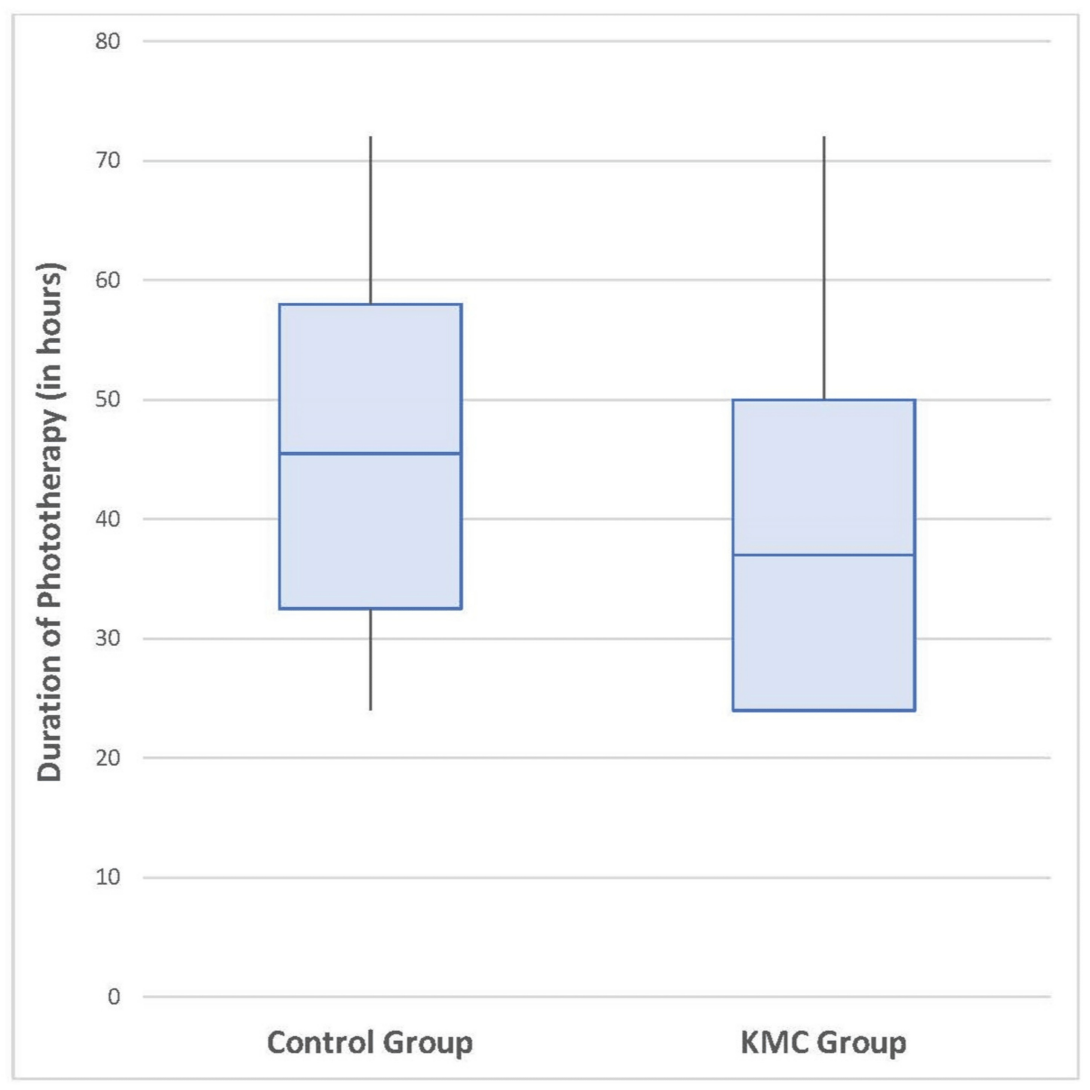
Box plot showing duration of phototherapy in the two groups KMC: kangaroo mother care

The mean duration of phototherapy was significantly higher in the control group (45.23 ± 16.74 hours) than in the KMC group (36.92 ± 18.2 hours) (p = 0.048), with a moderate effect size (Cohens d = 0.48). The median (IQR) duration of phototherapy in the control group was 45.5 hours (IQR: 32.5-58.0), and in the KMC group was 37.0 hours (IQR: 24.0-50.0) (Figure 2). Since the data were not normally distributed, the Mann-Whitney U test was also employed, resulting in a U value of 464 (p = 0.022).

The bilirubin levels were compared between the two groups at different time points. Initial peak bilirubin levels are similar (15.66 mg/dL vs. 15.82 mg/dL, p = 0.703) in both groups. The control group consistently showed higher levels than the KMC group (Table 2). The effect size indicated that KMC had a moderate and high effect at 24 and 48 hours, respectively, while at 72 hours, a small effect indicated that the outcome in both groups converged over time.

**Table 2:** Comparison of bilirubin levels at different intervals between the two groups KMC: kangaroo mother care Data presented as mean (SD), the t-test was used to calculate the p-value; p-value < 0.05 considered significant

Time	Control group (n = 26)	KMC group (n = 26)	t-value	p-value	Cohen’s d
At admission	15.66 (1.65)	15.8 (1.34)	5.68	0.703	-0.11
After 24 hours	13.2 (3.5)	11.7 (2.8)	4.92	0.047	0.47
After 48 hours	11.7 (2.5)	9.9 (1.8)	4.28	0.038	0.83
After 72 hours	10.0 (0.0)	9.9 (0.8)	3.67	0.773	0.18

## Discussion

This study aimed to investigate the impact of intermittent KMC on the duration of phototherapy needed for the resolution of NNH. A significant difference was observed between the two groups in the required phototherapy duration, with the KMC group needing 36.92 ± 18.2 hours, compared to 45.23 ± 16.74 hours in the control group (p = 0.048), with a moderate effect size. The period of phototherapy required in the KMC group was less than that in the control group. The results of the present study align with the findings of Samra et al., who reported that the combined use of phototherapy and KMC could reduce the length of hospital stay from 100 hours to 68 hours [13]. Previously, the effect of KMC during phototherapy on bilirubin profile was studied in preterm infants, using a fiberoptic phototherapy panel held against their back while receiving KMC, and a shallower descent was observed in the KMC group compared to the non-KMC phototherapy group, with a significant difference noted only on day 4 [15].

On the other hand, Goudarzvand et al. could not confirm the positive effect of KMC and phototherapy on reducing the bilirubin level and, consequently, the duration of phototherapy [16]. This discrepancy may be due to the short-term implementation of KMC in their study, where it was administered for one hour, divided into two 30-minute sessions during the evening shift over three consecutive days, while in the present study, the newborns received KMC at least three times a day for one hour with a mean duration of KMC 4.8 ± 2.0 hours, consistent with other studies [10,13,17], emphasizing the need to do KMC for a duration of at least over an hour to be effective. This may also highlight the plausibility of a dose-response relationship between the duration of KMC and the beneficial effects; however, further studies will be required to validate it.

In the present study, there was a statistically significant difference in bilirubin levels after 24 hours (p = 0.047) and after 48 hours (p = 0.038) between the two groups, suggesting that combining KMC with phototherapy is more effective in reducing bilirubin levels than phototherapy alone. Similarly, one study indicated that complementary measures like KMC or body massage with phototherapy decreased the serum bilirubin levels faster and reduced the length of phototherapy and hospitalization for term newborns compared to phototherapy alone [18]. It was consistent with the present study, further highlighting the potential benefits of integrating KMC with phototherapy in the management of neonatal jaundice and curtailing hospital stays. A meta-analysis concluded that the combination of KMC with phototherapy effectively reduced the serum bilirubin at 72 hours, and thus, the period of exposure to phototherapy and hospital stay was shortened [19]. The analysis noticed relatively large heterogeneity between the included studies in the duration of phototherapy in the KMC group, which could be because of the different study settings, ethnicities, NNH causes, adherence to protocols, discharge criteria, and healthcare factors.

Although phototherapy is a safe treatment option, conventional phototherapy devoid the baby of the benefits of skin-to-skin contact [5], whereas the combination of KMC with phototherapy offers this benefit, fulfilling the emotional needs of the mother-infant dyad and reducing the neonatal stress response. Early and uninterrupted skin-to-skin contact is essential for a newborn’s physical, emotional, and behavioral development [20]. Delays in this care can disrupt their natural instincts and bonding, making it harder for them to regulate their behavior and successfully engage in self-attachment and breastfeeding [21]. It plays a crucial role in reducing maternal stress and anxiety [22], minimizing neonatal stress responses, fostering positive maternal-infant interactions, and supporting breastfeeding.

Our study was limited by the sample size and by being a single center, which limits the applicability of the results to other centers. The inclusion of only healthy-term babies eludes the possibility of generalization of results to preterm and sick neonates. There could be a few confounding factors that may be missed due to a smaller sample size. Further multicenter studies, including preterm and sick neonates with a larger sample size, are recommended to confirm the findings and increase their generalizability and relevance. Long-term implications may also be studied. Still, implementing KMC alongside phototherapy has shown promise in reducing hospitalization costs by shortening stays and minimizing the need for intensive care.

## Conclusions

In conclusion, the present study highlighted the benefits of combining intermittent KMC with phototherapy in reducing treatment duration and improving bilirubin management outcomes in neonates with jaundice. These findings emphasize the potential for integrating KMC into standard care protocols to enhance treatment efficacy, reducing short-term and long-term adverse effects of phototherapy by reducing the duration and improving overall neonatal health outcomes. However, further large-scale multicentric randomized trials are needed to validate the findings. Overall, integrating KMC with phototherapy represents a holistic strategy that addresses both the clinical management of neonatal jaundice and the enhancement of maternal-infant interactions, thereby supporting comprehensive neonatal care. We thus suggest the promotion of KMC in neonates with hyperbilirubinemia.
